# Diagnostic Accuracy of Fecal Calprotectin for Predicting Relapse in Inflammatory Bowel Disease: A Meta-Analysis

**DOI:** 10.3390/jcm12031206

**Published:** 2023-02-02

**Authors:** Jin-Tong Shi, Nuo Chen, Jia Xu, Hemant Goyal, Zhi-Qi Wu, Jie-Xin Zhang, Hua-Guo Xu

**Affiliations:** 1 Department of Laboratory Medicine, The First Affiliated Hospital of Nanjing Medical University, Nanjing 210029, China; 2 Division of Gastroenterology, Hepatology & Nutrition, University of Texas Health Sciences Center, Houston, TX 77030, USA

**Keywords:** Fecal calprotectin, inflammatory bowel diseases, biomarker, diagnosis

## Abstract

Fecal calprotectin (FC) levels correlate with the disease activity of inflammatory bowel diseases (IBD); however, the utility of FC in predicting IBD relapse remains to be determined. We aim to evaluate the efficacy of fecal calprotectin in predicting the relapse of inflammatory bowel disease. We searched Pubmed (MEDLINE), Embase, Web of Science, and the Cochrane library databases up to 7 July 2021. Our study estimated the pooled sensitivity and specificity, summary receiver operating characteristic (SROC) curve, and the optimal cut-off value for predicting IBD relapse using a multiple threshold model. A total of 24 prospective studies were included in the meta-analysis. The optimal FC cut-off value was 152 μg/g. The pooled sensitivity and specificity of FC was 0.720 (0.528 to 0.856) and 0.740 (0.618 to 0.834), respectively. FC is a useful, non-invasive, and inexpensive biomarker for the early prediction of IBD relapse. An FC value of 152 μg/g is an ideal threshold to identify patients with a high relapse probability.

## 1. Introduction

Inflammatory bowel diseases (IBD) are chronic gastrointestinal disorders with a remitting and relapsing course and are associated with multiple complications. IBD incidence has increased in industrialized countries with increased healthcare expenditure and poor quality of life [1]. Ulcerative colitis (UC) and Crohn’s disease (CD) represent the two main types of IBD. Since the clinical course of IBD remains unpredictable, there is an urgent need to develop serum and fecal biomarkers to help predict relapse to take appropriate measures to reduce complications [2,3].

Endoscopy plays an essential role in the diagnosis, management, prognosis, and surveillance of IBD [4,5]. However, in routine practice, endoscopic evaluations of disease severity are relatively expensive and invasive. In addition, endoscopic monitoring is the least acceptable for of monitoring from the patients’ perspectives [6]. Accurate tests that are practical, non-invasive, and inexpensive would be ideal. Several promising serologic and fecal biomarkers have emerged that could fulfill this role, including fecal calprotectin (FC), C-reactive protein (CRP), and erythrocyte sedimentation rate (ESR) [7]. CRP and ESR are useful to confirm ongoing mucosal inflammation but are of less value to predict a future relapse since elevated levels of these markers have not been found to precede a clinical flare [7]. Furthermore, there is considerable heterogeneity in CRP generation based on the genetics of individual patients [8]. These limitations have encouraged the development of alternative tests, specifically stool biomarkers with higher specificity for intestinal inflammation.

FC is an excellent marker of intestinal inflammation. Calprotectin is a calcium and zinc-binding protein formed by a heteromeric complex of two subunits, S100A8 and S100A9. It is derived from human neutrophils and monocytes and represents around 60% of soluble cytosol proteins in human neutrophil granulocytes [9]. Calprotectin is a heterocomplex of the S100 proteins S100A8 and S100A9 (also called myeloid-related protein 8 and MRP14) [10]. It has been classically considered an abundant innate immune protein due to its antimicrobial activity depriving microorganisms of transition metals [11]. In addition, it has been associated with antiproliferative and immunomodulatory effects [12].

FC is currently incorporated as a routine test to aid in diagnosing and monitoring IBD [13]. Though increasing evidence has been published about the usefulness of FC in predicting IBD relapse, the optimal cut-off of FC has been controversial [14]. This systematic review and meta-analysis aimed to evaluate the efficacy of FC as a predictor of IBD relapse in adult patients in remission and to obtain a cut-off value to help in clinical practice.

## 2. Materials and Methods

### 2.1. Search Strategy

The meta-analysis followed the guidelines for Preferred Reporting Items for Systematic Reviews and Meta-Analyses (PRISMA) (Supplementary Table S1). We searched the articles published in Pubmed (MEDLINE), Embase, Web of Science, and the Cochrane library databases from inception until July 2021. The following search terms were used: “Calprotectin”, “IBD”, “UC”, and “CD”. The search strategy was noted in Supplementary Table S2. References of eligible articles were also screened. All meeting abstracts were excluded because of insufficient data to reconstruct the 2 × 2 table. We restricted our search to studies published in English only.

### 2.2. Study Selection

The eligible articles were initially screened independently by three reviewers (JTS, NC, and JX) based on their titles and abstracts. Full manuscripts of the potentially eligible articles were reviewed while removing the duplicates. Three reviewers (JTS, NC, and JX) independently assessed articles if they met the including criteria: ① diagnostic cohort studies, ② prospective studies using FC to predict IBD relapse, ③ FC level was measured at baseline, ④ patient’s baseline status was in remission, ⑤ sufficient data to reconstruct 2 × 2 table, ⑥ relapse was confirmed by clinical symptoms and endoscopic results, and ⑦ studies conducted on adult’s patients with IBD. All disagreements were resolved through discussion with the authors (HG and HGX). Studies that did not meet our prespecified criteria were excluded.

### 2.3. Data Extraction and Quality Assessment

Three reviewers (JTS, NC, and JX) extracted the following characteristics from each article independently: name of the study, country, authors, publication year, age, gender, FC cut-off value, data for the construction of 2 × 2 table, follow-up time, type of FC assay, reference standard, medical treatment, and funding sources. The quality of included articles was assessed by JX using the QUADAS-2 (A revised tool for the Quality Assessment of Diagnostic Accuracy Studies) [15]. All disagreements were resolved through discussion with three authors (JTS, NC, and JX). The specific criteria were explained in Supplementary Table S3.

### 2.4. Data Synthesis and Analysis

To obtain the summary receiver operating characteristic (SROC) curve and an optimal cut-off for predicting IBD relapse, we applied the multiple thresholds model, which included multiple cut-off values with the results of true positive, true negative, false positive, and false negative. The multiple threshold model is a new approach for the meta-analysis of diagnostic test accuracy studies where several studies reported more than one threshold and the corresponding sensitivity and specificity values. The approach is based on the idea of estimating the distribution functions of the biomarker with the nondiseased and diseased individuals using a common parametric assumption (normal or logistic) for the distribution of a continuous biomarker. This was achieved using a mixed effects model with the study as a random factor [16]. The optimal cut-off was defined as the point where the Youden index (sensitivity + specificity − 1) was maximized [17]. We used the inverse variance weight to measure the mean value in order to represent the weight of individual studies. The model that minimized the restricted maximum likelihood criterion was chosen as the best. In addition, we used random effects bivariate models to calculate pooled sensitivity and specificity, and the same is true for subgroup analysis. We also created forest plots for each study.

To explore the clinical utility of FC for the prediction of the relapse of IBD, we performed a Fagan nomogram. The relationship between the prior probability, the likelihood ratio, and the posterior test probability is portrayed graphically by comparing 25, 50, and 75% prior probabilities [18]. The likelihood ratios obtained represented three clinical application scenarios: ① low suspicion of relapse for IBD: 25%; ② high suspicion of relapse for IBD: 75%; and ③ worst-case scenario: 50%.

Additionally, we calculated the positive predictive values (PPVs) and negative predictive values (NPVs) related to different cut-off values under varying levels of relapse rate by using a linear mixed-effects model for multiple thresholds model [19].

Although a funnel plot is the basic graphical method to detect publication bias, it is not recommended to be used in the diagnostic meta-analysis because of the multiple thresholds, so we did not explore publication bias [20].

As the thresholds can vary for each study, it was essential to see how close the observed results are to the receiver operating characteristic (ROC) curve rather than how dispersed they are in the ROC space [21]. The magnitude of heterogeneity is best accessed by a graph, which can be observed by the dispersion of points and the closeness between the 95% prediction region and 95% confidence region in the SROC curve [22]. We performed subgroup analysis related to the type of diseases, follow-up time, reference standard, and FC assay.

All data analyses were performed using STATA version 15, “DIAGMETA” package of R language for windows (Version 3.6.0; R Foundation for Statistical Computing, Vienna, Austria) and MetaDisc version 1.4 [16,19,23].

## 3. Results

### 3.1. Selection, Characteristics, and Quality of Studies

**Figure 1:**
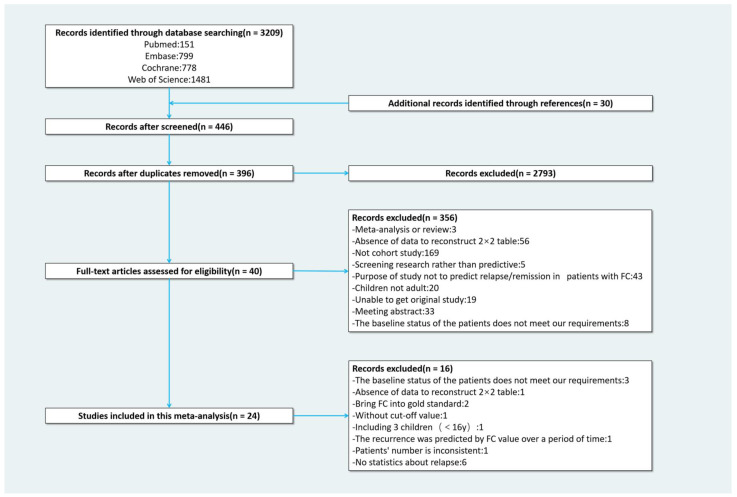
Flow chart of selection process.

Our initial search yielded 3209 papers. Additionally, we added 30 papers from the review of relevant literature references. After removing duplicates and screening titles and abstracts, 396 studies were selected for full-text review. Of these, 356 were initially excluded. After data extraction and discussion, another 16 studies were excluded. The reasons for the exclusion of each study were listed in the Table 1. Finally, 24 studies were included with a total of 2260 patients of whom 715 relapsed (Figure 1).

Table 2 summarizes the characteristics of the included studies. All studies used a prospective study design and enrolled patients with quiescent IBD at baseline. In included studies, 7/24 (29.2%) of them [40,41,42,43,44,45,46] solely involved patients with CD, while 7/24 (29.2%) of them [47,48,49,50,51,52,53] involved only patients with UC. The remaining 10/24 (41.7%) studies [7,54,55,56,57,58,59,60,61,62] included patients with both UC and CD. FC was measured at baseline. The IBD relapse was identified with clinical symptoms and/or endoscopic findings on follow-up over a period of time. The follow-up period varied between studies, as shown in Table 2. The definitions of relapse in each study were listed in the Table 3. Since the definition of recurrence varies from study to study, for the sake of analysis, we divided them into two broad categories: clinical relapse and endoscopic relapse. A total of 5/24 (20.8%) studies used endoscopy as a reference; 19/24 (79.2%) studies used clinical symptoms or therapy change. Cut-off values for predicting relapse ranged from 50 to 500 μg/g, and most of them were mainly in the range of 100–250 μg/g.

Overall, the quality of the included studies was good (see the results of QUADAS-2 in Supplementary Table S4). Eleven studies [42,44,45,47,48,51,52,53,56,58,62] did not mention whether the patients enrolled were consecutive or not. Blinding of reference standard results was reported in all but one study [61]. Four studies [46,47,50,51,61] reported the blinding of index test results, while others did not mention it.

### 3.2. Performance of FC at the Optimal Cut-Off Value

#### 3.2.1. Primary Outcome

**Figure 2:**
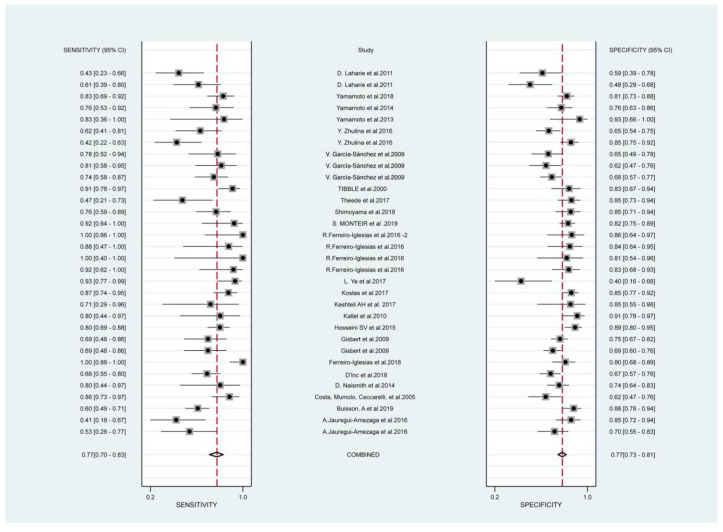
Forest plots of pooled sensitivity and 1 − specificity of Fecal Calprotectin at remission for predicting relapse in inflammatory bowel disease. Plots display diagnostic probabilities of included studies and their corresponding 95% confidence intervals [7,40,41,42,43,44,45,46,47,48,49,50,51,52,53,54,55,56,57,58,59,60,61,62].

**Figure 3:**
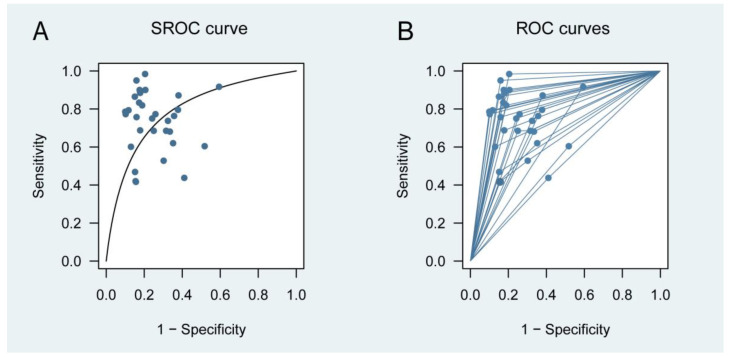
Test performance for predicting relapse in patients with IBD. (**A**) Multiple threshold SROC curve; (**B**) multiple threshold ROC curves based on the multiple thresholds model. Circles represent information on sensitivity and specificity. ROC, receiver operating characteristic; and SROC, summary receiver operating characteristic.

Figure 2 presents the forest plots of sensitivity (true positive rate) and 1 − specificity (false positive rate) for the 24 studies. Combining all available data from the 24 studies using the multiple thresholds model, the resulting SROC curve is shown in Figure 3. An optimal cut-off value of 152 μg/g was identified. At 152 μg/g, the Youden index reached its maximum (Supplementary Figure S1). Its corresponding sensitivities and specificities were 0.720 (0.528 to 0.856) and 0.740 (0.618 to 0.834), respectively. The area under the SROC curve (AUC) for predicting IBD relapse was found to be 0.794.

Furthermore, the bivariate model was also applied to evaluate the diagnostic performance of FC by using the data from just one cut-off reported for each study. Based on the multiple threshold model results, if a study reported multiple cut-off values, then the option closest to 152 μg/g was selected. The cut-off values ranged from 50 to 340 μg/g. Its corresponding sensitivities, specificities, and AUC were 0.80 (0.73 to 0.85), 0.78 (0.73 to 0.82), and 0.85 (0.82 to 0.88), respectively. The SROC for the bivariate model can be found in Supplementary Figure S2.

#### 3.2.2. Post-Test Probability of Relapse

**Figure 4:**
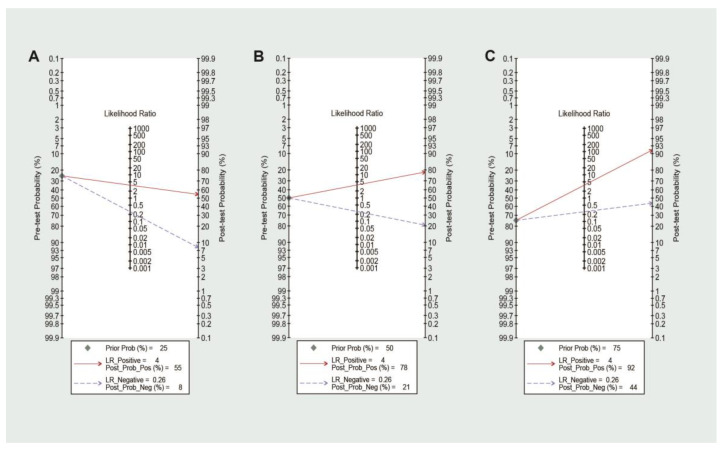
Fagan nomogram analysis evaluating the clinical utility of FC for predicting IBD relapse according to different pretest probabilities (prior). (**A**) Pretest probability = 25%; (**B**) pretest probability = 50%; and (**C**) pretest probability = 75%.

In clinical practice, there is a need to understand the probability that a patient with quiescent IBD will relapse or not when an FC test result exceeds a certain threshold. The PPV and NPV varied for various relapse rates of IBD because these are related to the disease prevalence. Therefore, it was addressed with a multiple thresholds model, with a calculation of PPVs and NPVs related to the optimal and other common cut-off values for different levels of relapse rate (Supplementary Table S5). Employing an FC threshold of 152 μg/g, the highest NPV of 0.98 was observed when using the test in a low-relapse rate setting, i.e., when the relapse rate was no more than 5%. The highest PPV of 0.893 was observed in a high-relapse rate setting (72.5%). To further improve the analysis of the predictive effect of FC on relapse with the threshold of 152 μg/g, we additionally calculated the post-test probability with three different levels of relapse rate. The Fagan nomogram showed that FC testing changed the post-test probability of IBD (Figure 4). In the low suspicion of IBD relapse, the results showed that a negative post-test probability of 8% could be considered sufficient to exclude the high possibility of relapse. On the other hand, in the high suspicion of IBD relapse, a positive post-test probability of 92% could be considered sufficient to warn of the relapse within 24 months.

### 3.3. Subgroups Analysis 

In order to determine whether disease types (CD or UC), follow-up time (<1 year or ≥1 year), reference standard (clinic or endoscopy), and FC-assay (BÜHLMANN fCAL^®^ ELISA, Calprest^®^ or Human Calprotectin ELISA Kit, Cell Sciences Inc., Newburyport, MA, USA) were sources of heterogeneity, we performed subgroup analyses. Analyses showed similar summary performance for all subgroups (Supplementary Table S6).

## 4. Discussion

Our meta-analysis aimed to obtain an ideal cut-off value for predicting IBD relapse suitable for clinical use. Although we intended to stratify patients with UC and CD and tried to calculate a threshold unique for CD and UC using the multiple threshold model, there were not enough studies. However, by performing further subgroup analysis, we found that the sensitivity and specificity results did not change when the disease type was stratified into UC vs. CD using the random-effects bivariate models. Thus, we calculated the cut-off value for the IBD group (including patients with CD and UC). It was found that the pooled sensitivity of FC is 0.720 (95% CI 0.528–0.856) and the pooled specificity is 0.740 (0.618–0.834) with an AUC of 0.794 at the cut-off value of 152 μg/g. The estimated DOR is 14, indicating that FC is a useful biomarker in predicting the relapse of IBD.

### 4.1. Implications of Key Findings

Heida A et al. [63] suggested that FC levels in remission should be used to predict recurrence trends in patients with IBD. FC is inexpensive and non-invasive and has better specificity than CRP. FC is remarkably stable in stools for up 7 days at room temperature, enabling sample collection at home even in patients’ remote locations. These characteristics of FC may make monitoring IBD patients convenient and practical. Based on our results, we suggest that 152 μg/g was an appropriate threshold for monitoring IBD. Patients with higher FC levels (>152 μg/g) should be warned of the possibility of relapse within 24 months.

We used the comprehensive GRADE approach [64] to examine the validity of our results (Table 4). Recognition of the accuracy of FC as a substitute for outcomes important to patients is central to this approach. Detection of FC levels to predict relapse of patients with IBD will be valuable only if the FC monitoring improves the care of patients with IBD. Therefore, we inferred from the pooled sensitivity and specificity for the effect of the FC test on patient monitoring for IBD relapse. The key question is whether the numbers of false negatives (cases that the risk of recurrence was underestimated) and false positives (cases that the risk of recurrence was overestimated) are acceptable in this context.

In a hypothetical population of 100 IBD adults in remission (given an overall recurrence rate of 25%), eighteen patients should increase the frequency of FC testing. Additionally, they need to be monitored continuously and, if necessary, endoscopically examined to confirm recurrence. Fifty-six percent of patients have a low risk of recurrence in the future and only need to be observed according to the original plan. FC testing reduces the psychological burden of those patients. Seven patients will be missed. A false negative FC result can delay determining the patient’s risk of recurrence and delay treatment. Nineteen percent will be diagnosed as false positives, which results in inconvenience and unnecessary financial expense. There will also be a certain amount of stress on the mental side. We also hope that when determining whether the FC results are reliable, the factors that will produce false positives and false negatives should be excluded (Table 5). Based on the comprehensive analysis, FC is still recommended to predict recurrence in patients with IBD.

### 4.2. Comparison with Other Reviews

Previous meta-analyses have evaluated the performance of FC for relapse in patients with IBD [64,65,66,67]. However, an ideal cut-off value for FC was never determined. This is the first meta-analysis evaluating the FC level and obtaining an excellent cut-off value to distinguish whether patients would relapse in the near future, which is more helpful for clinical practice. YS Tham et al. [67] suggested that an FC cut-off value of 150 µg/g is associated with optimal diagnostic accuracy for postoperative endoscopic recurrence in CD. However, the performance for an FC level of 135 μg/g was not examined. Although it was the optimal cut-off value for the largest cohort they included, the value appeared in only one cohort and was not sufficient to obtain a pooled performance. Li et al. [64] showed that in patients with UC, the accuracy of FC was better in studies with a cut-off of ≥150 µg/g but did not discuss the optimal cut-off value. However, we used a novel multiple threshold model to obtain the ideal cut-off value by maximizing the Youden index rather than comparing it with other cut-off values. Also, this is the first cut-off value that takes IBD as a whole, and subgroup analysis proves that the results of UC and CD are similar. Therefore, this cut-off value is more convenient to be used in clinical practice.

Additionally, the diagnostic performance results obtained by the multiple threshold model would be a little lower but more realistic than by traditional approaches. This is because the multiple threshold model uses all the available information and results in an estimation of the performance of the biomarker, which avoids the drawback of using a single cut-off value. Previous meta-analyses only used one pair of sensitivity and specificity per study, which may lead to an overestimation of the SROC curve because there would be cut-off selection bias, and the ‘optimal’ point would be generally chosen [68].

### 4.3. Limitations of Study

We would also like to report some of the limitations of this study. The reference standard for the diagnosis of IBD relapse is still controversial; however, the reference standards used in the included studies are currently recommended. Also, QUADAS-2 is not a quality assessment method for prognostic tests, but it is still the most suitable method. We have deleted some items that are not applicable, which may lead to some deviation in the results. Additionally, due to the lack of data, we could not perform a subgroup analysis of medications used in these patients. The threshold may vary slightly when faced with different patients, and sometimes clinicians need to consider the extent of involvement and past history of the patient on the basis of the given threshold. In addition, a recent study reported that FC may increase with age, even 3–4 times [13], which reminded us it is possible that cut-off values vary in different age groups. Despite these limits, our analysis is rigorous and will further increase interest in performing high-quality studies using FC in predicting relapse in patients with IBD.

## 5. Conclusions

In conclusion, regularly measuring FC levels in IBD remission is a useful tool for the early prediction of relapse. The FC value of 152 μg/g is an ideal threshold for identifying patients with a high probability of relapse, suggesting careful follow-up and adjusting medications. Moreover, this noninvasive monitoring method will be better received by the patients without any preparation for colonoscopies and with high sensitivity and specificity. Further prospective high-quality trials are needed to determine the optimal FC measurement interval and cut-off value for the FC trend. In addition, it will also be useful to study further the predictive performance of combining markers, such as CRP and FC, for the relapse of patients with IBD.

## Figures and Tables

**Table 1:** List of excluded studies and reasons for their exclusion.

	Author	Year	Title	Reason for Exclusion
1	Boschetti G	2015 [24]	Accuracies of serum and fecal S100 proteins (calprotectin and calgranulin C) to predict the response to TNF antagonists in patients with Crohn’s disease.	The baseline status of the patients does not meet our requirements.
2	Lasson A	2013 [25]	Fecal calprotectin levels predict the clinical course in patients with new onset of ulcerative colitis.	The baseline status of the patients does not meet our requirements.
3	Yamamoto T	2015 [26]	Consecutive monitoring of faecal calprotectin during mesalazine suppository therapy for active rectal inflammation in ulcerative colitis.	The baseline status of the patients does not meet our requirements.
4	Brooks AJ	2017 [27]	Outcome of elective withdrawal of anti-tumour necrosis factor-α therapy in patients with Crohn’s disease in established remission.	Absence of data to reconstruct 2 × 2 table.
5	De Vos M	2011 [28]	Fast and sharp decrease in calprotectin predicts remission by infliximab in anti-TNF naïve patients with ulcerative colitis.	Bring FC into gold standard.
6	Sollelis E	2019 [29]	Combined evaluation of biomarkers as predictor of maintained remission in Crohn’s disease.	Bring FC into gold standard.
7	Reinisch W	2019 [30]	Fecal Calprotectin Responses Following Induction Therapy With Vedolizumab in Moderate to Severe Ulcerative Colitis: A Post Hoc Analysis of GEMINI 1.	Without cut-off value.
8	Mooiweer E	2015 [31]	Low fecal calprotectin predicts sustained clinical remission in inflammatory bowel disease patients: a plea for deep remission.	Including children (<16 y).
9	Molander P	2014 [32]	Does fecal calprotectin predict short-term relapse after stopping TNFα-blocking agents in inflammatory bowel disease patients in deep remission?	The recurrence was predicted by FC value over a period of time.
10	Garcia-Planella E	2018 [33]	Serial semi-quantitative measurement of fecal calprotectin in patients with ulcerative colitis in remission.	Patients’ numbers are inconsistent.
11	Tursi A	2019 [34]	Vedolizumab is effective and safe in real-life treatment of inflammatory bowel diseases outpatients: A multicenter, observational study in primary inflammatory bowel disease centers.	No statistics about relapse.
12	Bertani L	2020 [35]	Fecal Calprotectin Predicts Mucosal Healing in Patients With Ulcerative Colitis Treated With Biological Therapies: A Prospective Study.	No statistics about relapse.
13	Bertani L	2020 [36]	Serum oncostatin M at baseline predicts mucosal healing in Crohn’s disease patients treated with infliximab.	No statistics about relapse.
14	Guidi L	2014 [37]	Faecal calprotectin assay after induction with anti-Tumour Necrosis Factor α agents in inflammatory bowel disease: Prediction of clinical response and mucosal healing at one year.	No statistics about relapse.
15	Beswick L	2018 [38]	Exploration of Predictive Biomarkers of Early Infliximab Response in Acute Severe Colitis: A Prospective Pilot Study.	No statistics about relapse.
16	Reinisch W	2020 [39]	Association of Biomarker Cutoffs and Endoscopic Outcomes in Crohn’s Disease: A Post Hoc Analysis From the CALM Study.	No statistics about relapse.

**Table 2:** Summary of findings and raw data of studies included in the meta-analysis.

Study	Country	Age	Male (%)	Disease Type (N)	Follow-Up Time	TP	FP	FN	TN	Cut-Off (μg/g)	Reference Standard	Medication	FC-Assay ELISA
A. Jauregui-Amezaga 2014 [47]	Spain	46 (13.6 relapse); 46 (15.9 no relapse)	46.88	UC (82)	12 (m)	9	14	8	33	100	1, 2, 4	Mixed	Cerba Internacional
						7	7	10	40	250			
Buisson 2019 [62]	America	36 (16.3)	50.63	IBD (160)	12 (m)	53	9	35	63	100	1	Mixed	Genova Diagnostics
F.Costa 2005 [54]	Italy		56.96	IBD (79)	12 (m)	30	17	4	28	150	1, 3	Mixed	Calprest
		41.2 (12.7)		UC (41)		17	4	2	18				
		35.7 (11.6)		CD (38)		13	13	2	10				
D. Naismith 2014 [40]	UK	18–83	35.87	CD (97)	12 (m)	8	21	2	61	240	2, 3, 4	Mixed	Buhlmann
D’Inca 2008 [55]	Italy	15–80	51.85	IBD (162)	12 (m)	39	35	18	70	130	1	Mixed	Calprest
				UC (97)		26	18	11	42				
				CD (65)		13	17	7	28				
Ferreiro-Iglesias 2018 [56]	Spain	18–78	49.47	IBD (106)	12 (m)	30	13	0	52	130	1	anti-TNF	Buhlmann
Gisbert 2009 [57]	Spain	43 (13)	51.30	IBD (163)	12 (m)	18	43	8	94	150	1	Mixed	PhiCal
						18	34	8	103	167			
				UC (74)		9	20	4	41	150			
						9	16	4	45	164			
				CD (89)		9	23	4	53	150			
						9	18	4	58	169			
Hosseini 2015 [48]	Iran	20–83	51.30	UC (157)	12 (m)	59	9	15	71	341	1, 3	NA	Buhlmann
Kallel 2010 [41]	Tunis	15–66	43.40	CD (53)	12 (m)	8	4	2	39	340	1, 3	Mixed	Calprest
Keshteli 2017 [49]	Canada	42.7 (14.8)	45.00	UC (20)	12 (m)	5	2	2	11	124	1	Mixed	Buhlmann
Kostas 2017 [58]	Greece	17–76	51.68	IBD (149)	6 (m)	41	15	6	87	261	1, 3	NA	Buhlmann
L. Ye 2017 [42]	China	24 (23–43.5 relapse); 28 (19–42.5no relapse)	64.52	CD (62)	24 (m)	27	9	2	6	225	1, 2, 3	Mixed	Buhlmann
Ferreiro-Iglesias 2016 [43]	Spain	18–68	47.17	IBD (53)	2 (m)	11	7	1	34	160	1	IFX	Buhlmann
				UC (20)	2 (m)	4	3	0	13	198			
				CD (33)	2 (m)	7	4	1	21	160			
R.Ferreiro-Iglesias 2016 [59]	Spain	24–64	43.33	CD (30)	4 (m)	9	3	0	18	204	1	ADA	Buhlmann
S. Monteir 2019 [44]	Portugal	38.4 (12.2)	45.83	CD (144)	6 (m)	12	23	1	108	327	2,3	Mixed	Buhlmann
Shimoyama 2018 [50]	Japan	NA	NA	UC (196)	12 (m)	26	7	8	39	114	1	Mixed	Cell Sciences
Theede 2017 [51]	Denmark	39.3 (13.92)	72.86	UC (70)	12 (m)	7	8	8	47	321	3	Mixed	Buhlmann
Tibble 2000 [7]	UK	33 (16–77 CD); 49 (21–72 UC)	45.00	IBD (80)	12 (m)	40	6	4	30	50	1, 3	Mixed	NA
V. García-Sánchez 2009 [60]	Spain		57.04	IBD (135)	12 (m)	29	31	10	65	150	1	Mixed	Calprest
		40.4(13.1)		UC (69)		17	18	4	30	120			
		36.9(9.2)		CD (66)		14	17	4	31	200			
Y. Zhulina 2016 [61]	Sweden	50 (42–61 relapse)/ 58 (41–64 no relapse)	23.08	IBD (130)	3 (m)	5	17	3	79	500	3	Mixed	Buhlmann
						6	37	2	59	250			
					12 (m)	10	12	14	68	500			
						15	28	9	52	250			
					24 (m)	11	11	26	56	500			
						18	25	19	42	250			
Yamamoto 2013 [45]	Japan	32 (1.6)	60.00	CD (20)	12 (m)	5	1	1	13	170	1	Mixed	Cell Sciences
Yamamoto 2014 [52]	Japan	35.1 (0.8)	61.25	UC (80)	12 (m)	16	14	5	45	170	1, 2	Mixed	Cell Sciences
Yamamoto 2018 [53]	Japan	35 (31–39)	61.59	UC (164)	12 (m)	38	22	8	96	115	1	Mixed	Cell Sciences
D. Laharie 2011 [46]	France	27(17–65 relapse); 31(15–68 no relapse)	32.00	CD (65)	38 (m)	14	14	9	13	130	1,3	Mixed	Buhlmann
						10	11	13	16	250			

Age: Median (IQR) or mean (SD) or age range; disease type (No of patients); TP: true positive; FP: false positive; FN: false negative; TN: true negative; NA: not available. PhiCal, Bühlmann, Cell Sciences, Calprest, Genova Diagnostics, and Cerba Internacional are different fecal calprotectin test kits. Reference standard: 1: Clinic; 2: Endoscopy; 3: change in therapy or surgery; and 4: histopathology.

**Table 3:** Definitions of relapse in each study included.

Study	Disease Type	Definition of Relapse
A. Jauregui-Amezaga 2014 [47]	UC	Presence of blood in stool and MES ≥ 3 with histologic confirmation.
Buisson 2019 [62]	UC	Reappearance of clinical manifestation (SCCAI > 2 with subscore > 1 for at least one item among stool frequency and rectal bleeding) leading to medication intensification, hospitalization, or colectomy.
	CD	Reappearance of clinical manifestation (HBI > 4) leading to therapeutic intensification, hospitalization, or CD-related surgery.
F.Costa 2005 [54]	UC	Worsening of symptoms, accompanied by an increase in the UCAI score to >4, sufficient to require a change in therapy (addition of steroids, immunosuppressors, surgery, etc.).
	CD	Worsening of symptoms, accompanied by an increase in the CDAI score to >150, sufficient to require a change in therapy (addition of steroids, immunosuppressors, surgery, etc.).
D. Naismith 2014 [40]	CD	An unplanned escalation in therapy, progression of disease phenotype by the Montreal classification or hospitalisation and/or emergency surgery for active CD.
D’Inca 2008 [55]	UC	ET scores exceeding 4 and requiring additional treatment.
	CD	CDAI exceeding 150, with an increment of more than 50 points over the baseline score (75 points in resected patients) and requiring additional treatment.
Ferreiro-Iglesias 2018 [56]	UC	PMS > 2.
	CD	HBI > 4.
Gisbert 2009 [57]	UC	Truelove modified index > 11 points.
	CD	CDAI > 150.
Hosseini 2015 [48]	UC	Elevated Seo activity index higher than 220 or worsening of symptoms (including abdominal pain, diarrhea with or without blood and rectal bleeding) sufficient to require a change in therapy (increasing the dose, changing the current drug (s), addition of steroids, hospitalization or surgery).
Kallel 2010 [41]	CD	CDAI > 150 or an increase of more than 100 from the inclusion value and was sufficiently severe to warrant treatment.
Keshteli 2017 [49]	UC	PMS ≥ 3.
Kostas 2017 [58]	UC	(1) Significant increase in respective clinical activity indices above accepted cut-offs for remission in UC (Simple Colitis Activity Index ≥ 3) and/or (2) step up in the patient’s therapeutic regimen, including surgery for intractable disease-related symptoms.
	CD	(1) Significant increase in respective clinical activity indices above accepted cut-offs for remission in CD (HBI ≥ 5) and/or (2) step up in the patient’s therapeutic regimen, including surgery for intractable disease-related symptoms.
L. Ye 2017 [42]	CD	Worsening symptoms requiring intensified therapy or surgery or a CDAI score > 150, with confirmation by ileocolonoscopy.
Ferreiro-Iglesias 2016 [43]	UC	PMS > 3.
	CD	HBI > 4.
R.Ferreiro-Iglesias 2016 [59]	CD	HBI > 4.
S. MONTEIR 2019 [44]	CD	An unexpected escalation in therapy, hospitalization or surgery for active CD with ileocolonoscopy and inflammatory activity assessed by SES-CD or Rutgeerts score.
Shimoyama 2018 [50]	UC	The sum of ‘stool frequency’ score (0–3) and ‘rectal bleeding’ score (0–3) in the Mayo scoring system exceeding 0.
Theede 2017 [51]	UC	The symptoms of active UC demanding adjustment of actual or initiation of new UC therapy.
Tibble 2000 [7]	UC	HBI > 4 and an increase of >2 from the inclusion value. All relapses were of sufficient severity to warrant a change in treatment.
	CD	CDAI > 150 with an increase of >100 from the inclusion value. All relapses were of sufficient severity to warrant a change in treatment.
V. García-Sánchez 2009 [60]	UC	Worsening of the symptoms, accompanied by or a modified TW score of ≥11 points.
	CD	Worsening of the symptoms, accompanied by a CDAI score of ≥150 points.
Y. Zhulina 2016 [61]	IBD	Increasing symptoms necessitating intensified medical therapy or surgery.
Yamamoto 2013 [45]	CD	CDAI > 150 with an increase of ≥70 points.
Yamamoto 2014 [52]	UC	Worsening of stool frequency and/or rectal bleeding with an endoscopic score of 2 or 3.
Yamamoto 2018 [53]	UC	Worsening of stool frequency and/or rectal bleeding with the MES of 2 or 3.
D. Laharie 2011 [46]	CD	Increasing symptoms (CDAI > 250 within 2 weeks or CDAI > 150 with an at least 70 points of increase as compared with CDAI at week 14) or the need for an additional steroid or IFX course or for a surgical resection.

HBI: Harvey-Bradshaw index; PMS: partial Mayo score; MES: Mayo endoscopic score; UCAI: ulcerative colitis activity index; CDAI: Crohn’s disease activity index; ET score: Edwards and Truelove (ET) scores; SCAI: Simple Colitis Activity Index; SES-CD: Simple Endoscopic Score for Crohn Disease; and IFX: Infliximab.

**Table 4:** Effect of pooled sensitivity and specificity of fecal calprotectin on patients.

Test Result	Number of Participants (Studies)	Number of Results per 100 Patients Tested (95% CI)			Importance (Grade) *	Comments
		Prevalence 25%	Prevalence 50%	Prevalence 75%		
True positive (TP)	2457 (24)	18 (13 to 21)	36 (26 to 43)	54 (40 to 64)	8	Benefit from early identification of relapse.
False negative (FN)		7 (4 to 12)	14 (7 to 24)	21 (11 to 35)	9	Detriment from delays in identification of relapse and treatment.
True negative (TN)		56 (46 to 63)	37 (31 to 42)	19 (15 to 21)	8	Benefit from reassurance and relief of economic costs.
False positive (FP)		19 (12 to 29)	13 (8 to 19)	6 (4 to 10)	7	Detriment from undertake unnecessary psychological burden and financial expenditure.

CI: Confidence interval. * GRADE recommends classifying patient-important outcomes on a 9-point scale: 7–9: critical for decision making; 4–6: essential but not critical for decision making; and 1–3: of lower importance to patients.

**Table 5:** Causes of abnormal results for fecal calprotectin other than inflammatory bowel disease.

Type of the Causes	Specific Reasons
Infections	Giardia lamblia
	Bacterial dysentery
	Viral gastroenteritis
	Helicobacter pylori gastritis
	Clostridium difficile
	HIV
Malignancies	Colorectal cancer
	Gastric carcinoma
	Intestinal lymphoma
	Pancreatic cancer
	Polyposis intestinalis
Drugs	NSAIDs
	PPIs
Other gastrointestinal diseases	Gastro-oesophageal reflux disease
	Cystic fibrosis
	Coeliac disease (untreated)
	Diverticular disease
	Protein losing enteropathy
	Colorectal adenoma
	Juvenile polyp
	Autoimmune enteropathy
	Microscopic colitis
	Liver cirrhosis
	Gastrointestinal bleeding
	IBS
	Proctitis after radiation therapy
	Colon inflammation bag
	Pancreatitis
Lifestyle	Obesity
	Physical inactivity
Age	<9 years
	>65 years
Others	Bowel preparation for colonoscopy
	Rheumatologic diseases
	Perianal disease
	Stoma
	Immune deficiency
	Intestinal transplant
	Proteolysis
	Food allergy (untreated)

HIV: human immunodeficiency virus; NSAIDs: nonsteroidal anti-inflammatory drugs; PPIs: proton pump inhibitors; and IBS: irritable bowel syndrome.

## Data Availability

This research is a meta-analysis and all data have been uploaded along with the Supplementary Materials.

## Supplementary Materials

The following supporting information can be downloaded at: https://www.mdpi.com/article/10.3390/jcm12031206/s1, Figure S1: Youden Index of FC for predicting IBD relapse; Figure S2: Receiver operating characteristic graph of fecal calprotectin test in fecal calprotectin at remission for predicting relapse in inflammatory bowel disease, with 95% confidence region and 95% prediction regions; Table S1: The PRISMA checklist; Table S2: The search strategies of four databases; Table S3: Specific criteria of QUADAS-2; Table S4: Quality assessment of included studies; Table S5: Calculated sensitivities and specificities at cut-offs of 160, 50, 150 in predicting relapse and their corresponding PPVs and NPVs for different prevalences using the multiple thresholds model; and Table S6: Assessment of diagnostic accuracy in subgroup analysis.
