# Supplementary material for: Diagnostic Accuracy of Fecal Calprotectin for Predicting Relapse in Inflammatory Bowel Disease: A Meta-Analysis
Source: J Clin Med. 2023 Feb 2;12(3):1206. doi: 10.3390/jcm12031206 (PMC9917450; doi:10.3390/jcm12031206)
Supplement: Supplementary file 1 [file jcm-12-01206-s001.zip › jcm-2188747-supplementary.pdf]

Supplemental materials

Figure of contents

Figure S1: Youden Index of FC for predicting IBD relapse.
Figure S2: Receiver operating characteristic graph of fecal calprotectin test in fecal calprotectin at remission for predicting relapse in inflammatory bowel disease, with 95% confidence region and 95% prediction regions.

Table of contents

Table S1: The PRISMA checklist
Table S2: The search strategies of four databases
Table S3: Specific criteria of QUADAS-2
Table S4: Quality assessment of included studies.
Table S5: Calculated sensitivities and specificities at cut-offs of 160, 50, 150 μg/g in predicting relapse and their corresponding PPVs and NPVs for different prevalences using the multiple thresholds model.
Table S6: Assessment of diagnostic accuracy in subgroup analysis disease

Supplementary Figure S1: Youden Index of FC for predicting IBD relapse.

The optimal cutoff value of FC for predicting IBD relapse could be confirmed in the Youden index curve as 152 μg/g using the multiple threshold model. The optimal cutoff score was depicted as a solid vertical line.

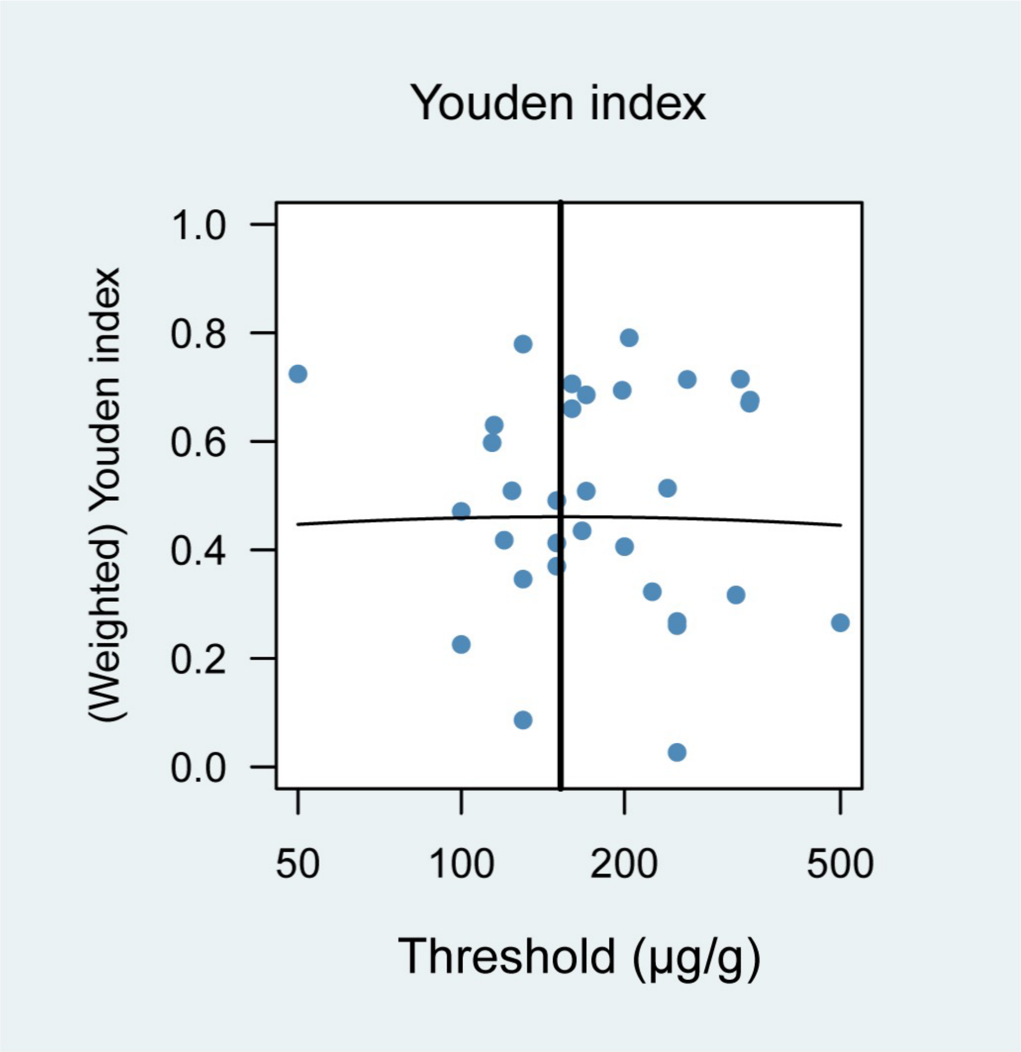

Supplementary Figure S2: Receiver operating characteristic graph of fecal calprotectin test in fecal calprotectin at remission for predicting relapse in inflammatory bowel disease, with 95% confidence region and 95% prediction regions. The confidence region consists of the most likely values of true summary sensitivity and specificity. It indicates the precision with which the summary points are estimated. The prediction region predicts the true sensitivity and specificity of a future study. The size of this region reflects the variation between studies.

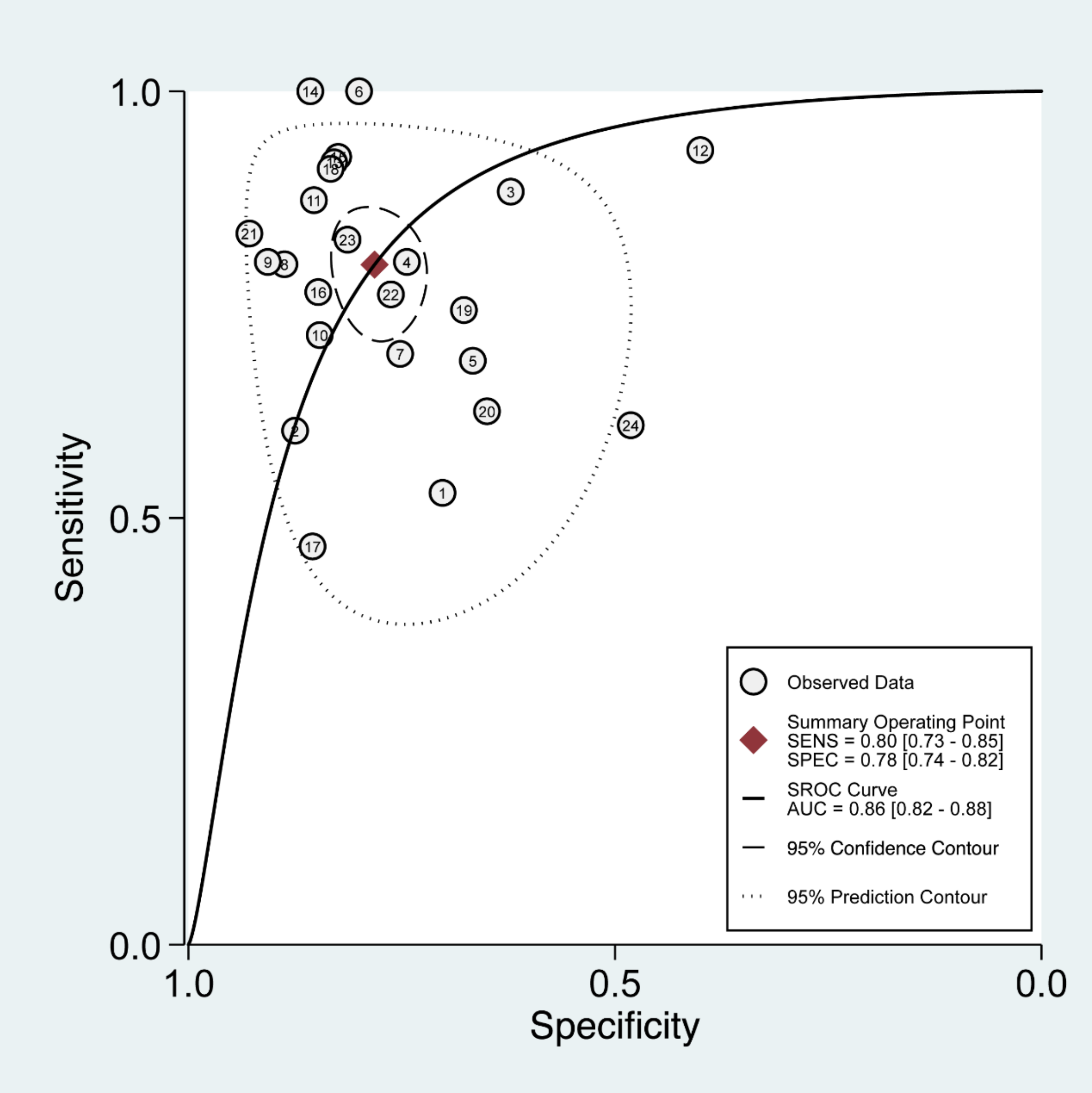

Supplementary Table S1: The PRISMA checklist

Section and Topic	Item #	Checklist item
**TITLE**		
Title	1	The report is identified as a meta-analysis.
**ABSTRACT**		
Abstract	2	The abstract includes Aims, Methods, Results, and Conclusions.
**INTRODUCTION**		
Rationale	3	Described in the Introduction.
Objectives	4	Stated in the Introduction.
**METHODS**		
Eligibility criteria	5	Stated in the Methods.
Information sources	6	Stated in the Methods.
Search strategy	7	Described in the Methods.
Selection process	8	Described in the Methods.
Data collection process	9	Described in the Methods.
Data items	10a	Described in the Methods.
	10b	Described in the Methods.
Study risk of bias assessment	11	Described in the Methods.
Effect measures	12	Described in the Methods.
Synthesis methods	13a	Described in the Methods.
	13b	Described in the Methods.
	13c	Described in the Methods.
	13d	Described in the Methods.
	13e	Described in the Methods.
	13f	Sensitivity analyses were not performed.
Reporting bias assessment	14	Described in the Methods.
Certainty assessment	15	Described in the Methods.
**RESULTS**		
Study selection	16a	Described in the Results.
	16b	Described in the Results.
Study characteristics	17	Described in the Results.
Risk of bias in studies	18	Presented in the Results.
Results of individual studies	19	Presented in the Results.
Results of syntheses	20a	Presented in the Results.
	20b	Presented in the Results.
	20c	Presented in the Results.
	20d	Sensitivity analyses were not performed.
Reporting biases	21	Presented in the Results.
Certainty of evidence	22	Presented in the Results.
**DISCUSSION**		

Section and Topic	Item #	Checklist item
Discussion	23a	Provided in the Discussion.
	23b	Discussed in the Discussion.
	23c	Discussed in the Discussion.
	23d	Discussed in the Discussion.
**OTHER INFORMATION**		
Registration and protocol	24a	The meta-analysis was not registered.
	24b	A protocol was not prepared.
	24c	No amendment to information provided.
Support	25	Described in the Funding Statement.
Competing interests	26	Declared in the Conflict-of-interest disclosure.
Availability of data, code and other materials	27	Reported in the Data Availability Statements.

Supplementary Table S2: The search strategies of four databases.

Database	Search strategy
Pubmed	(“Leukocyte L1 Antigen Complex”[Mesh] OR “calprotectin”[tw]) AND (“Inflammatory Bowel Diseases”[Mesh] OR “inflammatory bowel disease”[tw] OR “inflammatory bo wel diseases”[tw] OR “IBD”[tw] OR “Crohn”[tw] OR “Colitis”[tw])
Embase	(“calgranulin”/exp OR “calprotectin”/exp) AND (“enteritis”/exp OR “inflammatory bowel disease”/exp OR “inflammatory bowel diseases”/exp OR “ibd” OR “crohn” OR “colitis”/exp) AND [embase]/lim
Cochrane	#1 MeSH descriptor: [Leukocyte L1 Antigen Complex] explode all trees<br>#2 calprotectin or calgranulin<br>#3 #1 or #2<br>#4 MeSH descriptor: [Inflammatory Bowel Diseases] explode all trees<br>#5 inflammatory bowel disease or inflammatory bowel diseases or IBD or Crohn or Colitis<br>#6 #4 or #5<br>#7 #3 and #6
Web of Science	# 1 (TS=calprotectin) AND Document Type:(Article)<br># 2 (TS=Leukocyte L1 Antigen Complex) AND Document Type:(Article)<br># 3 (TS=calgranulin) AND Document Type:(Article)<br># 4 #1 OR #2 OR #3<br># 5 (TS=Inflammatory Bowel Diseases) AND Document Type:(Article)<br># 6 (TS=inflammatory bowel disease) AND Document Type:(Article)<br># 7 (TS=inflammatory bowel diseases) AND Document Type:(Article)<br># 8 (TS=Colitis) AND Document Type:(Article)<br># 9 (TS=Crohn) AND Document Type:(Article)<br># 10 #5 OR #6 OR #7 OR #8 OR #9<br># 11 #10 AND #4

Supplementary Table S3: Specific criteria of QUADAS-2

**Domain 1: Patients selection**	
Risk of Bias	***Could the Selection of Patients Have Introduced Bias?***
Signaling question 1	*Was a consecutive or random sample of patients enrolled?*<br>When studies enrolled eligible patients consecutively, we scored them as “yes”. Studies scored “unclear” if only the time range of enrollment was declared without the consecution mentioned. If studies did not mention any relevant information about enrolling patients, “no” would be scored.
Signaling question 2	*Was a case-control design avoided?*<br>The accuracy of diagnosis will be exaggerated when it comes to case-control trials. Under this circumstance, the studies were scored as “no”. If the studies avoided a case-control design, it would be scored as “yes”.
Signaling question 3	*Did the study avoid inappropriate exclusions?*<br>We scored the studies excluded patients inappropriately as “no”.
Applicability	***Are There Concerns That the Included Patients and Setting Do Not Match the Review Question?***<br>If patients were adults and in remission of inflammatory bowel disease based on their clinical symptoms and endoscopic results, we scored the studies as “low risk”. We scored studies as “high risk” that included patients with active or suspected inflammatory bowel disease. Studies that enrolled of healthy controls were also scored as “high risk”, because diagnostic accuracy may be exaggerated in this case.
**Domain 2: Index Test**	
Risk of Bias	***Could the Conduct or Interpretation of the Index Test Have Introduced Bias?***
Signaling question 1	*Were the index test results interpreted without knowledge of the results of the reference standard?*<br>Knowing the results of reference standard may influence the interpretation of index test results. But when the index tests were conducted before the reference standard, the potential risk of bias would be avoided. The aim of our study is to explore the baseline FC value for predicting IBD recurrence in a few months, we therefore acquiesced studies as “yes” unless stated otherwise.
Signaling question 2	*If a threshold was used, was it prespecified?*<br>Since our aim is to explore the FC threshold for predicting IBD recurrence, we deleted this item.
Applicability	***Are There Concerns That the Index Test, Its Conduct, or Its Interpretation Differ from the Review Question?***<br>In clinical practice, the commonly used detection method of FC is ELISA, such as BÜHLMANN fCAL® ELISA, Calprest® or Human Calprotectin ELISA Kit, Cell Sciences Inc., Massachusetts, USA and so on. In that case, we scored this item as low application concerns.
**Domain 3: Reference Standard**	
Risk of Bias	***Could the Reference Standard, Its Conduct, or Its Interpretation Have***

	Introduced Bias?
Signaling question 1	*Is the reference standard likely correctly classify the target condition?*<br>Up to now, there is not a definite gold standard for the prognosis of inflammatory bowel disease. However, the European Crohn's and Colitis Organization (ECCO) and the European Society of Gastrointestinal and Abdominal Radiology (ESGAR) published a guideline for diagnostic assessment in inflammatory bowel disease. To obtain a "yes", the studies must use the reference standard currently recommended. If not, we scored the study as "no".
Signaling question 2	*Were the reference standard results interpreted without knowledge of the results of the index test?*<br>The potential risk of bias is related to the previous knowledge about the results of the index test. When it was clear that the reference standard results interpreted without knowledge of the results of the index test, we scored this item as "yes". Studies scored as "unclear" if not illustrated.
Applicability	***Are There Concerns That the Target Condition as Defined by the Reference Standard Does Not Match the Question?***<br>The reference standard may be free of bias, but the target condition that it defines may differ from the target condition specified in the review question. As long as the target condition of the study was consistent with those of the reference standard, we scored the item as "low risk". Otherwise, it will be scored "high risk".
**Domain 4: Flow and Timing**	
Risk of Bias	***Could the Patients Flow Have Introduced Bias?***
Signaling question 1	*Was there an appropriate interval between index tests and the reference standard?*<br>As explained above, we deleted this item for the design of our research.
Signaling question 2	*Did all patients receive the same reference standard?*<br>When participants received different reference standards to confirm the diagnosis, verification bias would occur. We therefore scored studies that patients received the same reference standard as "yes" Otherwise, we scored as "no".
Signaling question 3	*Were all patients included in the analysis?*<br>Bias may exist when the number of patients enrolled is different from that in the 2×2 table. If the number is the same, we scored the studies as "yes". If not, we scored "no".

Supplementary Table S4: Quality assessment of included studies.

Study/Year	Risk of Bias				Applicability Concerns		
	Patient Selection	Index Test	Reference Standard	Flow and Timing	Patient Selection	Index Test	Reference Standard
A.Jauregui-Amezaga 2014 [47]	?	√	√	√	√	√	√
Buisson 2019 [62]	?	√	?	√	√	√	√
F. Costa 2005 [54]	√	√	?	√	√	√	√
D.Naismith 2014 [40]	√	√	?	√	√	√	√
D'Inca 2008 [55]	√	√	?	√	√	√	√
Ferreiro-Iglesias 2018 [56]	?	√	?	√	√	√	√
Gisbert 2009 [57]	√	√	?	√	√	√	√
Hosseini SV 2015 [48]	?	√	?	√	√	√	√
Kallel 2010 [41]	√	√	?	√	√	√	√
Keshteli AH 2017 [49]	√	√	?	√	√	√	√
Kostas 2017 [58]	?	√	?	√	√	√	√
L.Ye 2017 [42]	?	√	?	√	√	√	√
R.Ferreiro-Iglesias 2016 [43]	√	√	?	√	√	√	√
R.Ferreiro-Iglesias 2016-2 [59]	√	√	?	√	√	√	√
S.Monteir 2019 [44]	?	√	?	√	√	√	√
Shimoyama 2018 [50]	?	√	√	√	√	√	√
Theede 2016 [51]	?	√	√	√	√	√	√

Tibble 2000 [7]	√	√	?	√	√	√	√
V. García-Sánchez 2009 [60]	√	√	?	√	√	√	√
Y. Zhulina 2016 [61]	√	?	√	?	√	√	√
Yamamoto 2013 [45]	?	√	?	√	√	√	√
Yamamoto 2014 [52]	?	√	?	√	√	√	√
Yamamoto 2018 [53]	?	√	?	√	√	√	√
D.Laharie 2011 [46]	√	√	?	√	√	√	√

Supplementary Table S5: Calculated sensitivities and specificities at cut-offs of 160, 50, 150 μg/g in predicting relapse and their corresponding PPVs and NPVs for different prevalences using the multiple thresholds model.

Cut-off (μg/g)	Sensitivity	95% CI	Specificity	95% CI	Prevalence	PPV	NPV	FP*	FN*	FP-1000	FN-1000
160	0.72	0.52-0.85	0.74	0.62-0.84	0.05	0.13	0.98	24	1	243	14
					0.10	0.24	0.96	23	3	231	28
					0.20	0.41	0.91	20	6	205	57
					0.30	0.55	0.86	18	8	179	85
					0.40	0.65	0.80	15	11	154	113
					0.50	0.74	0.72	13	14	128	141
					0.60	0.81	0.64	10	17	102	170
					0.70	0.87	0.53	8	20	77	198
					0.72	0.88	0.50	7	21	70	205
50	0.79	0.57-0.92	0.66	0.46-0.81	0.05	0.11	0.98	33	1	327	10
					0.10	0.20	0.97	31	2	310	21
					0.20	0.36	0.93	28	4	275	42
					0.30	0.50	0.88	24	6	241	63
					0.40	0.61	0.82	21	8	207	83
					0.50	0.70	0.76	17	10	172	104
					0.60	0.78	0.68	14	13	138	125
					0.70	0.84	0.57	10	15	103	146
					0.72	0.86	0.54	9	15	95	151
150	0.72	0.53-0.86	0.74	0.62-0.83	0.05	0.13	0.98	25	1	248	14
					0.10	0.24	0.96	23	3	235	28
					0.20	0.41	0.91	21	6	208	56
					0.30	0.54	0.86	18	8	182	84
					0.40	0.65	0.80	16	11	156	111
					0.50	0.73	0.73	13	14	130	139
					0.60	0.81	0.64	10	17	104	167
					0.70	0.87	0.53	8	19	78	195
					0.72	0.88	0.50	7	20	72	202

FN, false negative; FP, false positive; NPV, negative predictive value; PPV, positive predictive value.
*Number of false positives and negatives in 100 hypothetical cases

Supplementary Table S6: Assessment of diagnostic accuracy in subgroup analysis.

Group	Comparison	Sen	Spe	PLR	NLR	DOR
**Disease types**	UC	0.75 [0.69,0.80]	0.78 [0.73,0.83]	3.4 [2.7,4.4]	0.32 [0.25,0.40]	11 [7,17]
	CD	0.82 [0.73,0.89]	0.72 [0.62,0.81]	3.0 [2.1,4.4]	0.24 [0.15,0.40]	12 [5,27]
**Follow-up time**	<1y	0.85 [0.71, 0.93]	0.77 [0.64, 0.86]	3.6 [2.1, 6.3]	0.20 [0.09, 0.44]	18 [5, 67]
	1y+>1y	0.78 [0.71, 0.84]	0.77 [0.72, 0.82]	3.4 [2.7, 4.3]	0.29 [0.21, 0.39]	12 [8, 19]
**Reference standard**	Endoscopic relapse	0.79 [0.61,0.90]	0.71 [0.58,0.82]	2.8 [2.0,3.9]	0.29 [0.16,0.54]	9 [5, 20]
	Clinical relapse	0.79 [0.72,0.85]	0.79 [0.74,0.84]	3.8 [2.9, 5.0]	0.26 [0.19,0.36]	15 [9,25]
**FC-assay**	Buhlmann	0.85 [0.73, 0.92]	0.78 [0.70, 0.84]	3.8 [2.7, 5.4]	0.20 [0.11, 0.37]	19 [8, 46]
	Calprest	0.77 [0.65, 0.85]	0.72 [0.60, 0.82]	2.8 [1.8, 4.3]	0.33 [0.20, 0.52]	8 [4, 19]
	Cell Sciences	0.79 [0.71, 0.86]	0.81 [0.76, 0.86]	4.3 [3.2, 5.7]	0.25 [0.17, 0.37]	17 [10, 30]
